# The Role of Machine Learning in Spine Surgery: The Future Is Now

**DOI:** 10.3389/fsurg.2020.00054

**Published:** 2020-08-21

**Authors:** Michael Chang, Jose A. Canseco, Kristen J. Nicholson, Neil Patel, Alexander R. Vaccaro

**Affiliations:** ^1^Department of Orthopaedic Surgery, Thomas Jefferson University, Philadelphia, PA, United States; ^2^Rothman Orthopaedic Institute, Philadelphia, PA, United States

**Keywords:** machine learning, deep learning, artificial intelligence, spine surgery, orthopedic surgery

## Abstract

The recent influx of machine learning centered investigations in the spine surgery literature has led to increased enthusiasm as to the prospect of using artificial intelligence to create clinical decision support tools, optimize postoperative outcomes, and improve technologies used in the operating room. However, the methodology underlying machine learning in spine research is often overlooked as the subject matter is quite novel and may be foreign to practicing spine surgeons. Improper application of machine learning is a significant bioethics challenge, given the potential consequences of over- or underestimating the results of such studies for clinical decision-making processes. Proper peer review of these publications requires a baseline familiarity of the language associated with machine learning, and how it differs from classical statistical analyses. This narrative review first introduces the overall field of machine learning and its role in artificial intelligence, and defines basic terminology. In addition, common modalities for applying machine learning, including classification and regression decision trees, support vector machines, and artificial neural networks are examined in the context of examples gathered from the spine literature. Lastly, the ethical challenges associated with adapting machine learning for research related to patient care, as well as future perspectives on the potential use of machine learning in spine surgery, are discussed specifically.

## Introduction

In clinical medicine, the rise of machine learning applications represents a new era of solving healthcare problems. This is particularly true in spine surgery where algorithmic decision support tools, computer assisted navigation, and surgical robots are already being used in the clinic and operating room. While the appetite for machine learning and its role in artificial intelligence has grown amongst spine surgeons, very little discussion has revolved around how to evaluate these applications and their contributions to patient care. In 2019 alone, 82 publications (more than twice the previous year) were PubMed indexed when searching for the terms “machine,” “learning,” and “spine” together. A core component of proper peer-review requires familiarity with machine learning methodology among clinicians. Until this can be achieved, machine learning in the spine literature will either foster skepticism or flawed enthusiasm. The intricacies and real patient-safety concerns when dealing with the spine necessitates that clinicians familiarize themselves with the terminology and guiding principles of machine learning. This review will introduce the origins of the artificial intelligence field and provide an organic discussion on how to practically synthesize machine learning modalities in spine surgery. A glossary of key terms in this review can be referred to in Table 1.

**Table 1:** Glossary of key machine learning terminology.

**Terminology**	**Definition**
Artificial neural networks:	Deep machine learning inspired by the biological neural network of an animal brain and Hebbian learning (1).
Black box:	A short-term ethical challenge in machine learning where the process by which the computer reaches an outcome is not easily interpretable and is hidden from consumers and engineers alike (2).
Decision tree learning:	A supervised machine that visually resembles a tree with nodes, branches, and leaves. Trees are adept at identifying clusters of homogenous variables and predicting outcomes. Most commonly a classification and regression tree (3).
Deep learning:	Computers that utilize representation learning or hidden layers to characterize unlabeled input variables without much manual human engineering. Commonly used for natural language processing, self-driving automobiles, pharmaceutical drug research, among others (1).
Distributional shift:	A short-term ethical challenge in machine learning where the training dataset poorly represents the true test set, secondary to racial or socioeconomic biases, or outdated information (4).
Feature values:	Individual characteristics or variables that are associated with the outcome of interest. Feature engineering can either be manually conducted or automated (5).
Hebbian theory:	Based on neuropsychology work by Dr. Donald O. Hebb from his book, *The Organization of Behavior*. Dr. Hebb's work on neuronal plasticity contributed greatly to the initial architecture of artificial neurons and networks (6).
Insensitivity to impact:	An ethical challenge in machine learning where the algorithm is unaware of the consequences of a false-positive or false-negative test (4).
Linear classification:	A task that involves predicting categorical outcomes (i.e., type of fruit or species of animal).
Linear regression	A task that involves predicting discrete or numeric outcomes that are integers or serial numbers (i.e., patient reported outcome scores).
Machine learning:	The study of using algorithms and mathematics to predict outcomes or accomplish tasks with little instruction or explicit programming. A subset of artificial intelligence (7).
Reward hacking:	A long-term ethical challenge of machine learning where algorithms self-learn how to maximize favorable outcomes but do so by circumventing rules or cheating the system (4).
Supervised learning:	Learner attempts to describe the input-output relationship based on input variables that are labeled and have a grounded truth (5).
Support vector machine:	A machine learning modality that can either solve classification tasks by creating a maximum margin hyperplane between two outcomes, or regression tasks by plotting a best-fit plane. Involves significant human engineering through kernel functions to transform data into higher dimensions (8).
Unsupervised learning:	Learner attempts to describe the input-output relationship based on input variables that are unlabeled. Typically associated with deep learning (9).

## A Brief History of Artificial Intelligence and Data Science

The study of artificial intelligence (AI) originated back in the summer of 1956 when Dr. John McCarthy and contemporaries gathered at Dartmouth College. They “proceeded on the basis of the conjecture that every aspect of learning or any other feature of intelligence could in principle be so precisely described that a machine could be made to simulate it (10, 11).” While this meeting of great minds was significant, progress within AI has been undulating, with great successes followed by even greater failures. Notwithstanding, the recent establishment of larger data sets (or Big Data) has enabled scientists to overcome previous obstacles. During the advent of popularized AI in the 1980's, ~1% of humankind's information was available digitally. Presently, digital information technology accounts for 99% of data, which is estimated to be 5 zettabytes (5 × 10^21^ bytes) (12, 13). This amount of information is greater than the sum total if one were to store genomes from every person on Earth (1 × 10^19^ bytes) (14). At an individual level, one can appreciate the abundance of data stored in the cloud and the expansion of stored memory on a smartphone. Over the last decade, the United States healthcare system has also benefited from the Health Information Technology for Economic and Clinical Health (HITECH) Act, which spurred the adoption of electronic medical records (15). Experts have speculated that society is rapidly approaching a point where the totality of data eclipses what can be extracted from nature itself (12). This massive amount of data has also been bolstered by large-scale commercialization of computing hardware, particularly graphics processing units or GPU (16). This increased accessibility of GPUs has allowed researchers to complete largescale machine learning tasks even at home, a feat unachievable in previous decades. Modern society is at a crossroads where we have access to inordinate amounts of data and hardware, but little guidance on how to extract meaningful information that is applicable to everyday life.

## Overview of Machine Learning

**Machine Learning (ML)** is a subset of AI that focuses on developing automated computer systems (*learners*) that predict outputs through algorithms and mathematics (7). The output represents the machine's interpretation of complex relationships that may be either linear or non-linear. Performance is graded according to its level of *discrimination* (probability of predicting outcomes accurately) and *calibration* (degree of over- or under-estimating the predicted vs. true outcome) (17). Examples of ML applications encountered by spine surgeons include image classification [i.e., automated detection of vertebral body compression fractures on CT or MRI (18–20)], preoperative risk stratification models, clinical decision support tools (21–25), among others. The purpose of this review is to define basic ML terminology, discuss the difference between ML and classical statistics, detail common ML models, and introduce examples in spine research. A summary of included references to machine learning applications in spine surgery and research are shown in Table 2.

**Table 2:** Summary of machine learning applications in this review.

**Authors**	**Model(s)**	**Cohort**	**Type of outcome**	**Results**
Burns et al. (18)	SVM	150 CT scans	Vertebral compression fractures	SVM achieved sensitivity of 98.7% with a false-positive rate of 0.29.
Hoffman et al. (26)	SVM	27 cervical myelopathy patients	Postoperative ODI score (regression)	SVM was more accurate than multivariate linear regression for postoperative ODI.
Hopkins et al. (27)	DNN	4,046 posterior spinal fusions	Surgical site infections	Neural network employed 35 input variables with a model AUC of 0.79.
Hopkins et al. (28)	DNN	23,264 posterior spinal fusions	30-day readmissions	Neural network AUC of 0.81. ACS NSQIP database study.
Karhade et al. (23)	ANN, BPM^†^, CART, SVM	1,790 cases of spinal metastatic disease	30-day postoperative mortality	Although the neural network had superior discrimination, the Bayes Point Machine was more calibrated and accurate overall.
Khan et al. (29)	CART, GAM^†^, MARS^†^, PLS^†^, RF, SVM	173 cervical myelopathy patients	SF-36	GBM and Earth models achieved AUC between 0.74 and 0.77 for predicting improvement in PCS-36 over the MCID.
Mehta and Sebro (30)	SVM	370 DEXA scans	Lumbar fracture	SVM detected incidental lumbar fractures on DEXA with an AUC of 0.93 and over 94% sensitivity and specificity.
Ogink et al. (22)	ANN, BDT^†^, BPM^†^, SVM	28,600 lumbar surgery patients	Non-home discharge	Neural network had the highest degree of discrimination and calibration. ACS NSQIP database study.
Seoud et al. (31)	SVM	97 adolescents with scoliosis	Scoliosis classification (C1, C2 C3)	100 surface topography measurements per patient. SVM with one-against-all strategy predicted 72% of cases.
Stopa et al. (21)	ANN	144 lumbar surgery patients	Non-home discharge	External validation of ANN developed by Ogink et al. validation AUC was 0.89 with 0.50 PPV and 0.97 NPV.
Tee et al. (32)	CART	806 traumatic spinal cord injury patients	Cluster analysis	Internal nodes included AIS grade, AOSpine injury morphology, anatomical region, and age. Six clusters were identified.
Vania et al. (33)	CNN	32 CT scans	Spine segmentation	Outcomes included spine, background, and two masking or redundant classifications. Sensitivity and specificity of the algorithm were above 96%.
Varghese et al. (34)	CART	27 pedicle screw pullout conditions	Pedicle screw pullout failure	Three input variables included foam density, screw depth, and screw angle. Correlation between observed and predicted pullout events was 0.99.

*ANN, artificial neural networks; BPM, Bayes point machines; BDT, boosted decision trees; CART, classification and regression decision trees; CNN, convolutional neural networks; DNN, deep neural networks; GAM, generalized additive models; MARS, multivariable adaptive regression splines; PLS, partial least squares; RF, random forests; SVM, support vector machines. †Indicates machine learning modalities not discussed in this review*.

### Machine Learning Terminology

The two major forms of ML are supervised and unsupervised learning. **Supervised learning** entails labeled data based on a grounded truth (1, 5). For example, a database of lateral x-rays has films prelabeled as either “fracture” or “no fracture.” A portion of this data (**training** dataset) is analyzed to build a model that synthesizes the pattern between independent variables (i.e., pixel in an image) and dependent variables (presence or absence of pathology). Individual radiograph pixels in this example are known as **feature values** or **vectors** (1, 5). The remainder of the x-ray films (**untrained** dataset) are fed to the machine, which is then assessed based on its ability to accurately predict a fracture or otherwise. As such, supervised learning excels in exercises of *linear classification* (where the outputs are discretely defined categories) or *linear regression* (where the outputs are continuous values).

**Figure 1:**
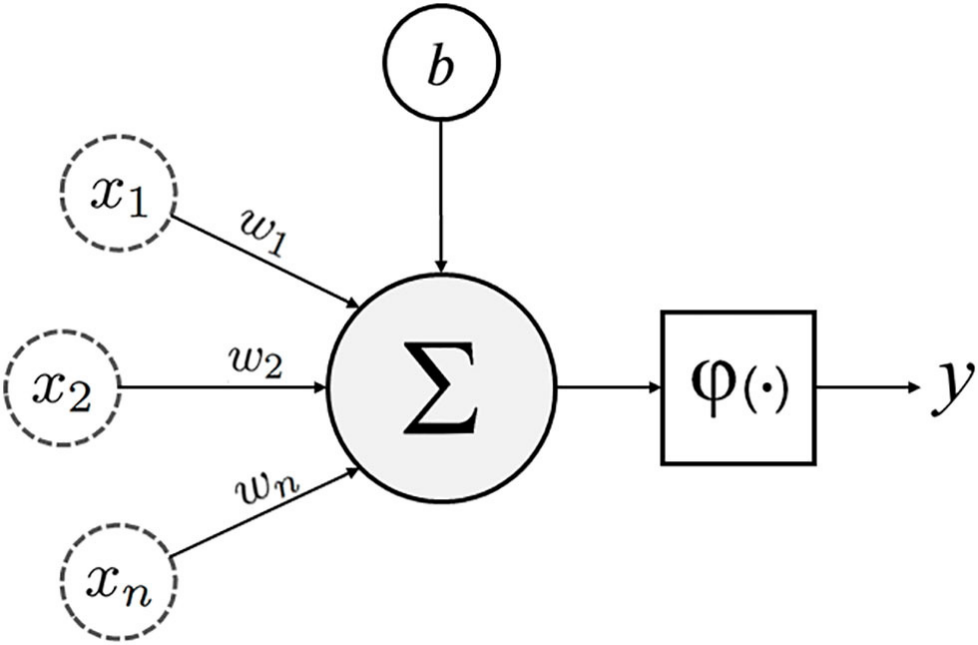
Schematic of an artificial neuron with inputs (x_1, 2…*n*_), weights (w_1, 2…*n*_), bias (b), transfer function (∑), activation function (φ) and output (y).

**Unsupervised learning**, on the other hand, involves the analysis of unlabeled datasets, and stems from neuropsychology research conducted by Dr. Donald Olding Hebb (1, 9). **Hebbian theory** describes the general framework (Figure 1) of neurons and their synapses, which enable humans and other animals to learn relationships and store memories (6). The proposition being that among the multitudes of neurons in the brain, it is the distinct synaptic connections between neurons and their repetitive firing that enable learning (6). Unsupervised machines (like humans) can appreciate non-linear relationships and do so without presumptions related to the data. Unsupervised learners are particularly adept at identifying clusters of related variables, detecting anomalies, and constructing **artificial neural networks** (detailed later) (1, 35). While unsupervised learning is thought to be the standard for the future, most current ML examples in spine surgery and clinical medicine are of the supervised variety.

### Machine Learning vs. Classical Statistics

The delineation between machine learning and classical statistics is quite nebulous because learners are built upon statistical modeling (Table 3). Both modalities also rely on robust preprocessing of data that is representative of the general population. However, whereas statistics emerged from the field of mathematics, ML emerged from computer science. For purposes of simplification, the two concepts can be differentiated by the type of question needed to be answered. Classical statistics *infers* relationships between variables, while ML attempts to *predict* these relationships (36, 37). Inference (or statistics) involves testing the null vs. alternative hypothesis for an effect with a measurement of confidence. Prediction (or machine learning) involves forecasting outcomes without demanding as to why resultant relationships exist. It is also essential to highlight that while ML may appear to be more advanced than statistical analysis, neither is superior and both should be considered for predictive modeling.

**Table 3:** Classical Statistics vs. Machine Learning.

**Classical statistics**	**Machine learning**
(1) Originates from mathematics	(1) Originates from computer science
(2) Inferring relationships	(2) Building algorithms
(3) Quantifying uncertainty	(3) Predicting outcomes
(4) High degree of manual programming	(4) Learns from experience - less programming
(5) One model at a time	(5) Multiple models in parallel

To illustrate this further, a research question might ask, “What risk factors are associated with non-routine discharge after lumbar decompression and/or fusion?” In fact, multiple studies using classical statistics have already implicated that patients' age, diabetes status, cardiovascular comorbidities, functional status, among others, all contribute to non-routine discharge (38–40). And with the expertise from practicing physicians, we can reason and clarify these findings. But translating these results in a clinical setting is complex, because it is unclear how one weighs the importance of each variable when optimizing patients preoperatively. ML enables the development of tools that allow surgeons to plug-in variables and generate probabilities of a non-routine discharge. Ogink et al. recently developed learners to predict discharge to a rehabilitation or skilled nursing facility after surgery for lumbar stenosis using the American College of Surgeons National Surgical Quality Improvement Program (ACS-NSQIP) database (22). They built multiple models in parallel and ultimately arrived at a neural network that achieved high levels of discrimination and calibration with an Area Under the Curve (AUC) of 0.74 from a Receiver Operating Characteristics curve (22). This tool has since been externally validated in a smaller cohort, where 97% of patients were accurately predicted to return to home after elective lumbar surgery (21). Such algorithms warrant further independent validation, but they allow for synthesizing unwieldly large datasets in a practical way. Above all, the purpose of machine learning is *performance* based on indiscriminate analysis. But when practicing medicine, the ability of a learner to predict outcomes accurately must also take into consideration *how* and *why* it reaches such conclusions. This controversy of applying ML clinically is colloquially termed the *black box*, which will be discussed at the end of this review.

## Popular Models for Machine Learning

With some basic ML terminology outlined, it is imperative that practicing physicians understand the architecture of learners encountered in peer-reviewed journals. Using examples from the spine literature, three ML modalities applicable to medicine will be discussed: (1) decision tree learning, (2) support vector machines, and (3) artificial neural networks. It is important to consider that while the following descriptions attempt to neatly categorize each model, they are flexible and can be adapted according to their needs. For example, support vector machines are often described as supervised models for linear classification, but there are many examples of them being used for unsupervised learning and non-linear classification exercises.

### Decision Tree Learning

**Figure 2:**
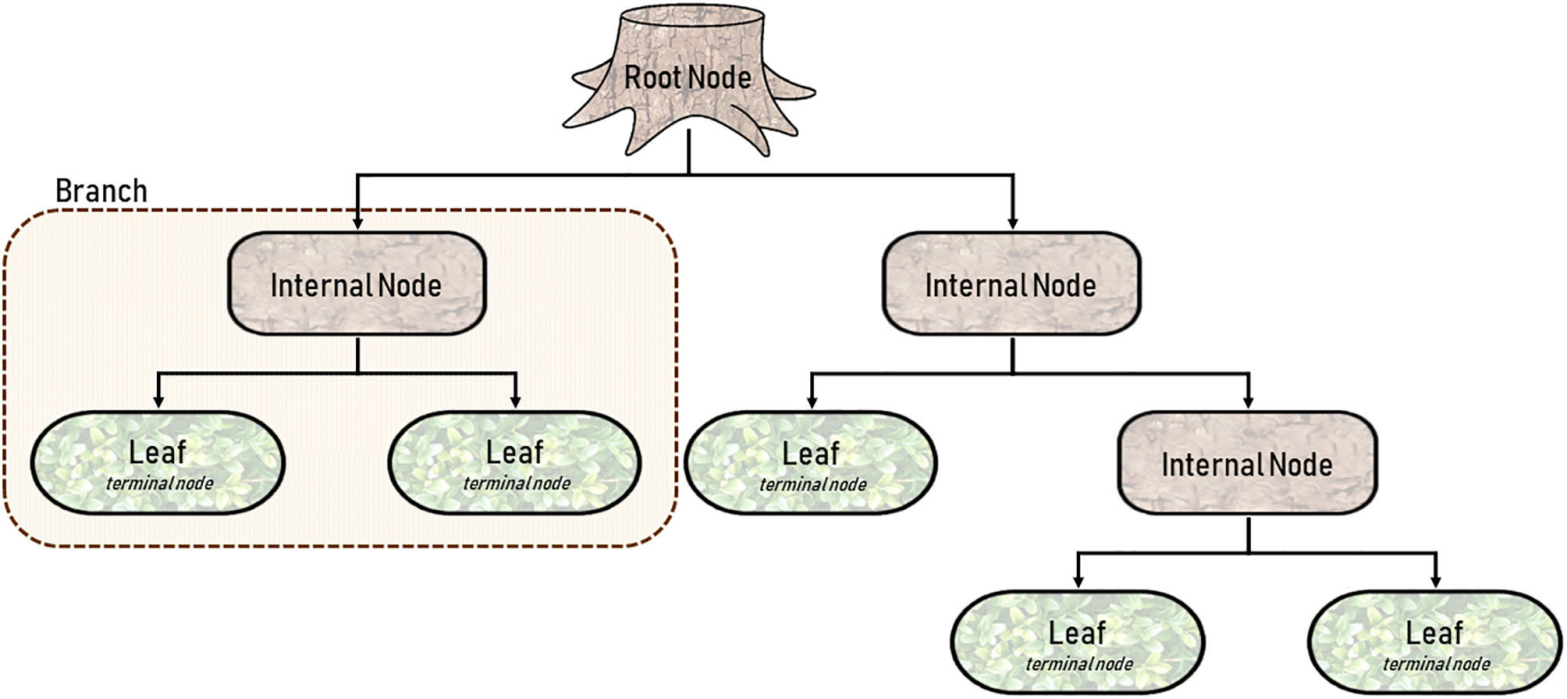
Diagram of a single Classification and Regression Tree (CART) with five terminal nodes or leaves.

**Decision tree learning**, or more specifically, **Classification and Regression Trees (CART)** is one of the more straightforward modalities because it is better appreciated visually, rather than mathematically (3, 37, 41). By definition, a CART can analyze variables that are either categorical (classification) or continuous (regression). As shown in Figure 2, a CART is an upside-down tree with three major components (1) internal nodes, (2) branches, and (3) leaves (3, 41). **Internal nodes** are conditions by which the learner evaluates or measures variables. **Branches** are the decisions derived from each node. And **leaves** (or **terminal nodes**) represent ends of the tree where an output is finalized. The figure depicted is simplistic, and in a real-world application would only represent a branch of a much larger CART. But decision trees have a habit of becoming unnecessarily *deep* or involving too many layers of complexity. Trees with excessive internal nodes sub-divide data into too many small clusters, such that the outcomes are grouped in a way that are practically meaningless. A CART is a fundamentally *greedy algorithm* because it naturally satisfies the condition at each node, rather than optimizing conditions across the length of the tree (3). **Pruning**, as the name suggests, allows for incremental improvements in the tree by eliminating conditions that are less important. It is relevant here to discuss the concept of *fit* in both classical statistics and machine learning (42). An **underfitting** model has no utility because it poorly approximates potential relationships (**Figure 5F**). On the other hand, an **overfitting** model attempts to observe the smallest of associations making the model relevant only to the training dataset, and by consequence, poorly generalizable (**Figure 5E**) (42). In other words, overfitting learners pay too much attention to the noise in the dataset. Pruning and other adjustments are necessary to minimize overfitting and to limit the complexity of the tree, all the while optimizing accuracy.

**Figure 3:**
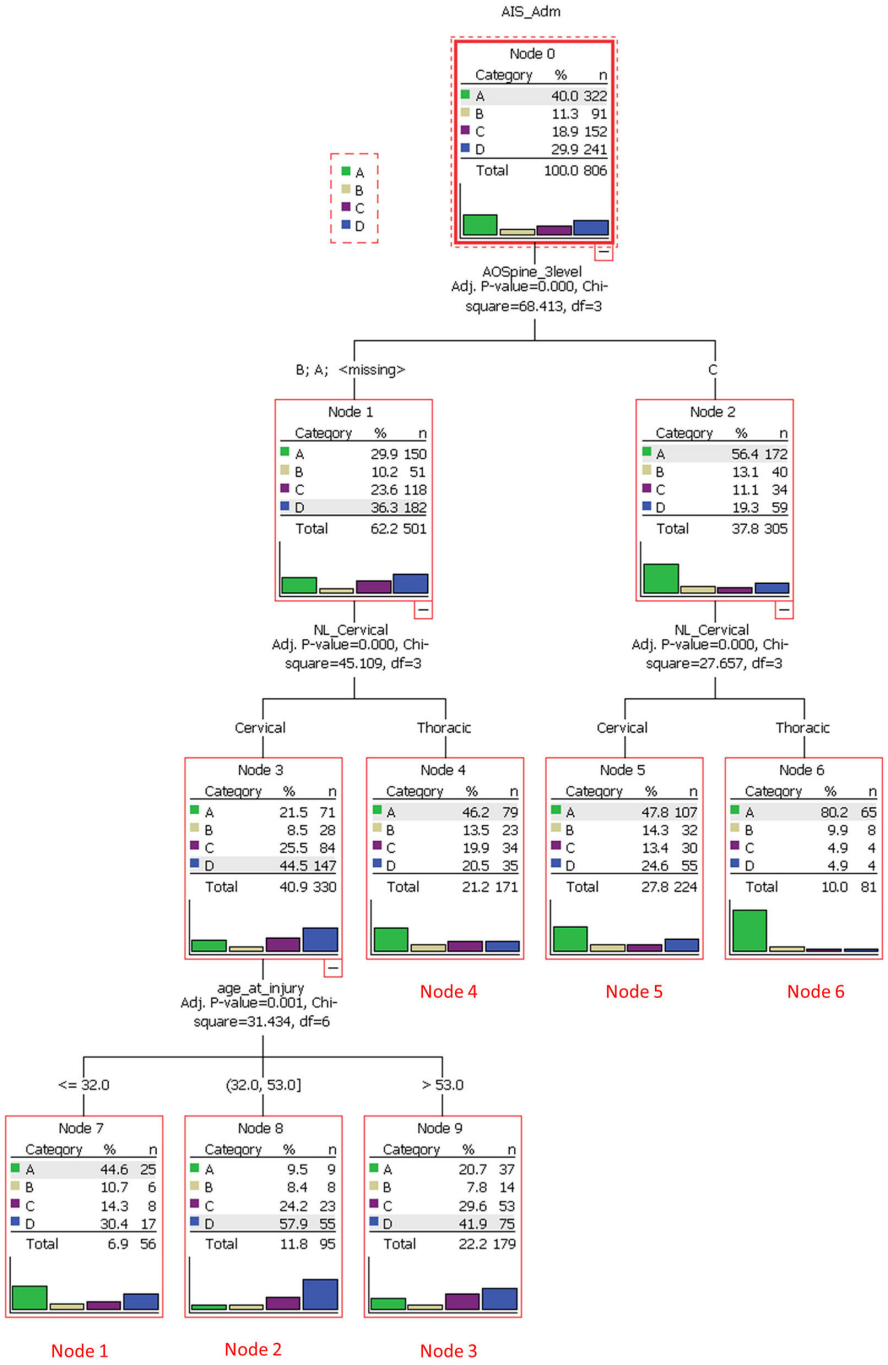
A decision tree analysis to stratify spinal cord injury cases and to identify clusters of homogeneous patients that would respond similarly to treatment. The root node was based on the American Spinal Injury Association Impairment Scale (AIS), which ranged from grade A through D. The subsequent internal node was based on AOSpine injury classification (class A/B or C). Each branch then underwent another node based on anatomical region (cervical or thoracic). Class A/B cervical injuries were divided further based on age. Six unique terminal nodes or clusters were identified. Reproduced with permission by Tee et al. (32).

Tee et al. application of decision tree learning for optimizing patient risk stratification after spinal cord injury provides a framework for understanding this modality (32). They combined different methods of assessing spinal cord function after trauma, including the American Spinal Injury Association (ASIA) Impairment Scale, total motor score (TMS) and the AOSpine classification system, to allow a decision tree to identify patient *clusters* that respond differently to treatment. As show in Figure 3, the cohort was first divided based on “ASIA grading (A-D)” (root node) and then evaluated at the first internal node, “AOSpine: A (compression), B (tension-band), or C (translational).” Interestingly, the learner concluded that it would be more worthwhile for the branches to keep A and B classifications together and C separate. The next internal node for each branch was binary, “cervical or thoracic injuries.” At this level in the tree, three leaves (nodes 4, 5, and 6) were finalized as these clusters were considered homogenous enough and not worth sub-dividing further. For example, node 5 represents AOSpine C injuries in the cervical region, whereas node 6 represents AOSpine C injuries in the thoracic region. Finally, the branch containing AOSpine A and B injuries in the cervical spine were passed through another internal node for “age,” generating another three leaves or clusters. The final six clusters are detailed in Table 4 (32). The results of this study provide a platform for external validation studies with other patient cohorts to compare this unique classification system with current ones. Tee et al. findings exemplify machine learning's ability to synthesize a multitude of variables that may associate non-linearly into a more easily digestible format. It is especially noteworthy that the investigators assembled a relatively large cohort of 806 patients for model building, a practice that is inconsistently applied in the spine literature.

**Table 4:** Final cluster analysis of spinal cord injury classifications based on decision tree learning.

**Node**	**AOSC type**	**Level of injury**	**Age at injury (years)**
1	A or B	Cervical	≤32
2	A or B	Cervical	>32–53
3	A or B	Cervical	>53
4	A or B	Thoracic	NA
5	C	Cervical	NA
6	C	Thoracic	NA

*Reproduced with permission by Tee et al. (32)*.

*AOSC, AOSpine injury morphology classification; NA, not applicable*.

The need for substantial patient datasets in spine surgery is particularly noticeable when exploring ML applications for predicting patient-reported outcomes. Exploratory investigations using decision tree learning have been pursued in spine research. Khan et al. utilized seven different supervised learners to predict improvement in SF-36 (PCS/MCS) scores after surgery for degenerative cervical myelopathy (29). The architecture of their model included multiple comorbidities, physical exam findings, imaging, baseline characteristics, among others. They set the minimal clinically important difference or MCID at +4.0 points for both PCS and MCS components of the SF-36. All seven learners were similarly accurate for predicting MCS improvement postoperatively, including their CART with an AUC of 0.74. However, no learner was particularly better than logistic regression (AUC 0.71), and the performance of the PCS model was by comparison poor. Moving forward, it is likely that the spine literature will be inundated with publications running multiple statistical and ML models in parallel for comparative analysis. And while Khan et al. pilot investigation provides a framework for understanding machine learning, their sample size (130 training, 43 testing) leaves some concern as to the generalizability of the findings. The relationship between the natural history of spinal pathology, surgical interventions, and postoperative outcomes is delicate; and the proper use of ML for describing these relationships will require a multicenter and multidisciplinary effort to coalesce massive patient databases.

**Figure 4:**
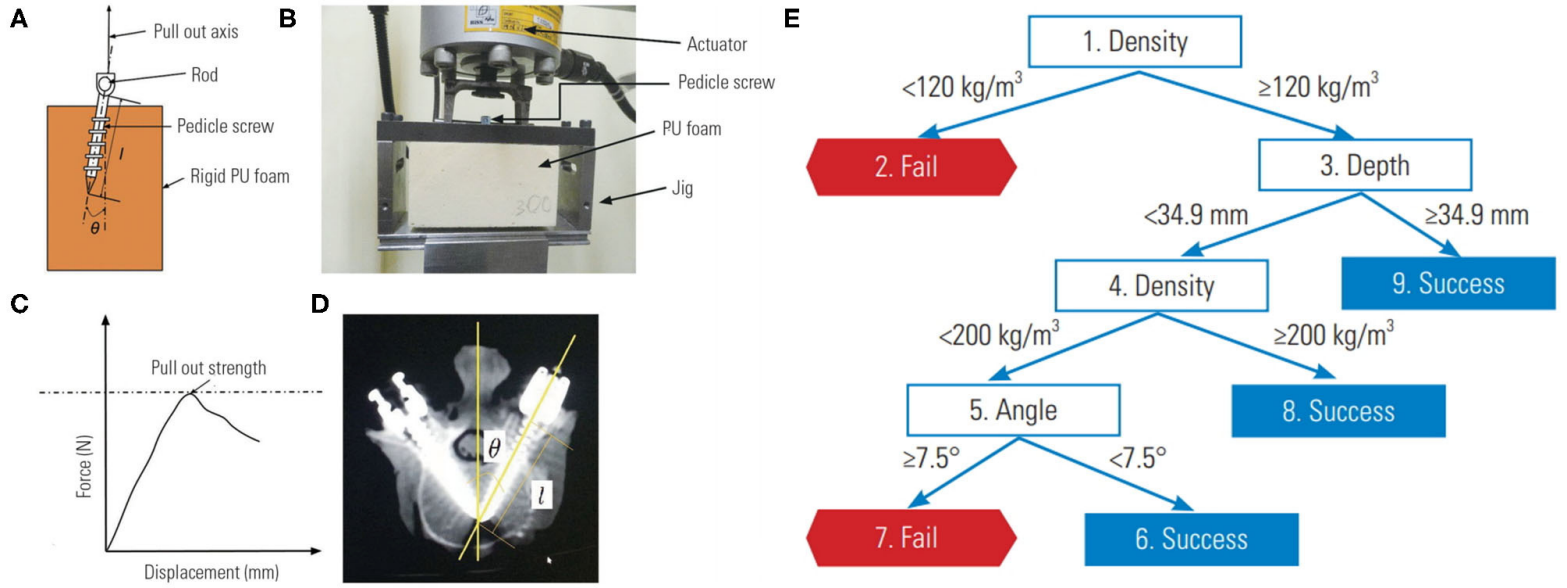
Biomechanical model for testing pedicle screw pullout-strength. **(A)** Schematic of rigid polyurethane foam to mimic normal, osteoporotic, and extremely osteoporotic bone. **(B)** Apparatus to test pedicle screw pull-out. **(C)** Force vs. displacement graph from pullout studies. **(D)** Anatomy of pedicle screw instrumentation. **(E)** Decision tree learning to predict pedicle screw pullout success vs. failure in relation to foam density, screw depth and insertion angle. Reproduced with permission by Varghese et al. (34).

Lastly, decision tree learning can also help with characterizing medical device performance. Varghese and colleagues, using their own pedicle screw pullout strength protocol, showed that ML could be used to synthesize problems that have a large number of input permutations (34, 43). Their investigation involved the use of differing foam densities to mimic normal, osteoporotic, and extremely osteoporotic bone (Figure 4A). An actuator apparatus would then insert pedicle screws into the foam at three insertion angles, and three insertion depths (Figure 4B) (43). In total, 27 (3^3^) permutations of these variables were analyzed using four separate models to determine pullout failure (<650 Newtons of force) or success (≥650 Newtons of force) (Figure 4C). Varghese et al. produced a promising model with very low error rates and an AUC of 1.00 for predicting pedicle screw failure, which was internally validated against a separate set of novel permutations (i.e., different pedicle screw insertion angles and foam densities) (**Figure 6**) (34). Their best learner was actually a **random forest regression**, which like a CART, is a subtype of decision tree analysis (41). As the name suggests, random forests sample random batches of the data, form multiple trees, and then combine the findings to construct a singular tree. Random forests minimize overfitting and other biases by employing the *Law of Large Numbers*, such that the average of multiple trees is more accurate than a single tree. The final decision tree constructed for pedicle screw pullout failure is shown in Figure 4E.

### Support Vector Machines

**Figure 5:**
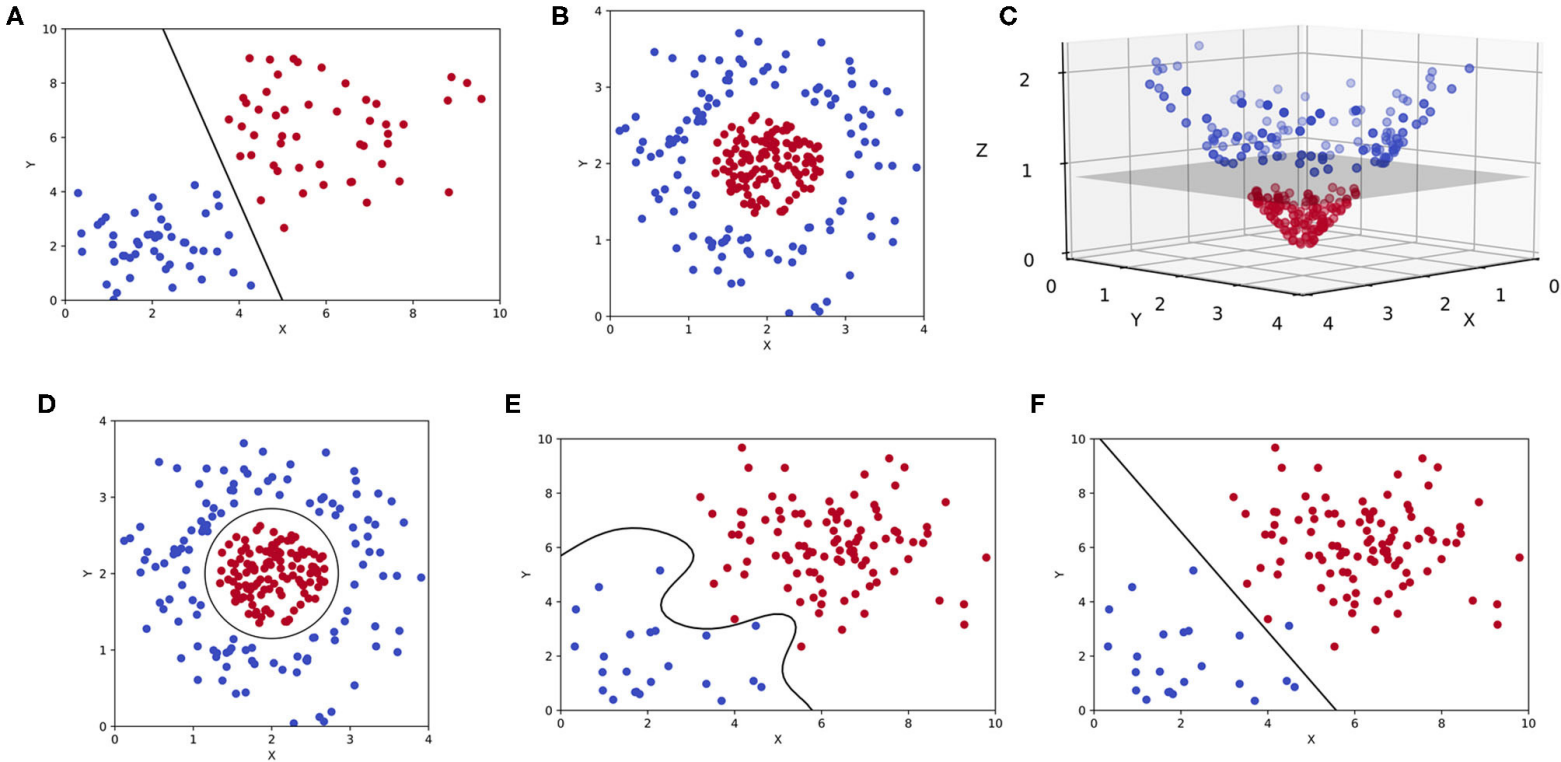
Support vector machines (SVMs). **(A)** Two-dimensional representation of a binary classification problem in an SVM represented by two features (x, y) with a maximum-margin hyperplane. **(B)** The same binary classification problem as before, but the outcomes are linearly inseparable in two-dimensions. **(C)** A linear kernel is used to transform the previous plot, allowing for a hyperplane to be constructed in three-dimensions (x, y, z). **(D)** A two-dimensional representation of the same hyperplane created in higher dimensions. **(E)** An overfitting SVM that is being influenced by outlier data or noise. **(F)** An underfitting SVM that fails to maximize the distance between two outcomes in a binary classification task.

Support vector machines (SVMs) are also a commonly encountered ML modality in clinical literature. SVMs are intuitive and best appreciated graphically as shown in Figure 5. Although comparable to CARTs in exercises of linear classification or regression, SVMs accomplish such goals by constructing a **hyperplane** (8, 44). For a classification exercise, the hyperplane represents a line (or plane) that maximizes the distance between two categorical outcomes, which is also known as a *maximum-margin* hyperplane (Figure 5A). But as one can appreciate in Figure 5B, not all two-dimensional representations of data (only “x” and “y” coordinates) can be separated linearly with a hyperplane in that same dimension. Often, mathematical transformations or **kernel functions** are needed to transform the data into a *higher* dimension (44). As shown in Figure 5C, the same dataset plotted in three dimensions (3D) can be easily separated by a hyperplane. This transformation is prototypical and involves the inclusion of a “z” coordinate that equates the product of x and y, such that each outcome is plotted in 3D as (x,y,z) or (x,y,x^*^y). This is also known as a *linear kernel*. The byproduct of an SVM for an otherwise linearly inseparable dataset is shown in Figure 5D, where higher dimensional hyperplanes are represented as a circle in lower dimensions. However, like pruning, kernels can be overly extrapolated leading to overfitting and generating sub-clusters of outcomes that are incidental and practically meaningless (Figure 5E).

Delving into the literature, SVMs are popular for classifying and detecting the presence of spine pathology on imaging. For example, a common problem when managing patients with osteoporosis arises from missed fractures on routine DEXA (Dual-Energy X-Ray Absorptiometry) scans (30). Given separate management guidelines for osteoporotic patients with and without fractures, Mehta and Sebro developed a model to detect incidental lumbar spine fractures from a large cohort of routine DEXA scans (30). The two outcomes or classifiers were “control” and “fracture.” The input variables to characterize the model included baseline demographics and ancillary data from the DEXA scan (i.e., bone mineral density, Z-scores, T-scores, among others). They conducted four SVMs in parallel, using different types of kernel functions, but ultimately arrived at a linear kernel with a high AUC of 0.93 against the training set, and an AUC of 0.90 against the test set (30). Their investigation exemplifies the potential of ML for automated detection of pathology. Such innovation can minimize missed diagnoses that are critical to quality care, especially in this case for incidental lumbar fractures on routine DEXA, where the error rate has been reported to be as high as 15.8% (45).

Another example of an image classification task achieved through SVMs was conducted by Seoud et al. The investigators attempted to determine scoliosis curve based on a modified Lenke classification system (C1, C2, or C3) for adolescents by analyzing surface topography data captured by multiple cameras (31). As a learning point, this is an example of applying SVMs with 3 outcomes (or classifications) instead of two. Seoud and colleagues addressed this problem by opting for a “one-against-all” approach, where the model compares C1 scoliosis curves to C2/C3 curves (46). And as discussed previously, the learner finds the ideal dimension where the outcomes can be linearly separated with the largest margin of distance between points. In this example, an overfitting model would be one where the SVM describes sub-clusters of scoliosis classifications that are clinically irrelevant. Seoud et al. model for classification based on topography alone accurately predicted over 72% of cases (31).

In addition to image classification tasks, SVMs have also been applied for predicting outcomes after spine surgery. Hoffman et al. prospectively evaluated patients undergoing surgery for degenerative cervical myelopathy, and attempted to predict postoperative outcomes including Oswestry Disability Index (ODI), modified Japanese Orthopedic Association scale (mJOA), and handgrip pressure (26). Their model illustrated how SVMs can also be used for regression. In contrast to classification, support vector *regressions* involve hyperplanes that *minimize* the distance between variables because the goal is to predict a continuous variable rather than a discrete one. Hoffman and colleagues also constrained the model to three input variables in order to curtail overfitting, which included preoperative ODI, symptom duration, and handgrip pressure. When compared to a traditional multiple linear regression, they achieved a higher goodness-of-fit or *R*^2^ of 0.93 via the SVM (26). While the prospective study design was a strength, the cohort was limited to only 20 patients. Herein lies the perpetual conflict between statistical power and generalizability when using ML. Models for predicting risk necessitate prospective data, but the feasibility of large datasets is limited to national databases, which are likely heterogeneous and retrospective.

Overall, SVMs are well-suited for general purpose machine learning (particularly in medicine) because tuning kernels allows for clinicians to assign appropriate weights according to their knowledge in that field (8). SVMs are also excellent tools for problems dealing with *high dimensional* data where the number of features far exceeds the number of observations or samples (47). Common examples of high dimensional data in clinical medicine include baseline demographics, preoperative risk factors, or gene expression levels. However, if the separation between two outcomes is unclear within a reasonable number of dimensions, SVMs struggle. And because SVMs are overly reliant on finely tuned kernels, the resultant models are only applicable to solving single problems (i.e., tools for predicting outcomes for cervical vs. lumbar surgery have to be separately and manually engineered). Counterintuitive to what has been discussed, SVMs are not proficient with very large data sets where the number of observations far exceed features (opposite of high dimensional data). As the number of points or samples increase, so does the noise, generating far too many outliers above and below the hyperplane (48).

### Artificial Neural Networks

**Figure 6:**
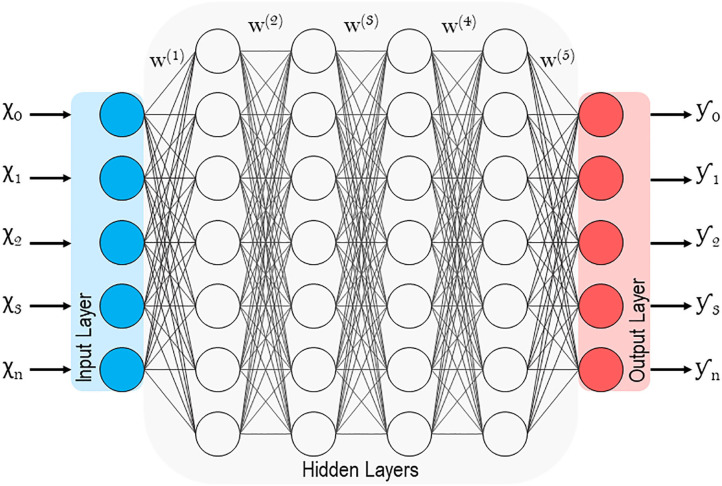
Schematic of a deep artificial neural network with multiple hidden layers.

Lastly, **Artificial neural networks (ANNs)** are of particular interest because they are associated with **deep learning**, which has been traditionally unsupervised (1, 49, 50). Supervised models, as discussed previously, involve feature values that are highly discriminatory because they have been meticulously engineered with intricate knowledge of the subject matter (in this case spine surgery) (48). Deep learning circumvents this through **representation learning**, where the learner automatically classifies raw unlabeled data (51). With minimal human engineering, these unsupervised learners generate highly discriminatory feature extractors that characterize the input-output relationship, while ignoring irrelevant variations. Like in Figure 6, ANNs extrapolate the single neuron construct in Hebbian learning into an entire network where **hidden layers** or *intermediate representations* help refine the network of input-output synapses between artificial neurons (1). For a more technical and in-depth review of deep learning and ANNs please refer to the work by Emmert-Streib et al. (52)

**Figure 7:**
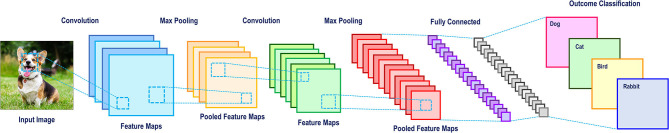
A convolutional neural network schematic for image classification.

ANNs are adept in computer vision (53–56), natural language processing (51, 57), and predicting downstream effects of genetic mutations (58–60). Computer vision is of interest to spine surgeons as it may potentially increase the efficiency and accuracy of reporting patient imaging. The classical computer vision task is identifying a “dog” in a photo (**Figure 9**). Manually extracting features is near impossible because no two photos of dogs are the same. Practically, humans recognize dogs in photos despite variations in their pose, environment, lighting or orientation of the photo, among others. However, machines can only interpret pixels in an image, none of which are specific to a dog. **Convolutional neural networks (CNNs)**, with the help of multiple hidden layers, are particularly adept at computer vision tasks and can be visualized graphically in Figure 7. The first hidden layer in a CNN *convolves* or filters the native input, extracting the “important” information and generates a **feature map** (a representation of the input). Subsequent **max-pooling** reduces complexity and minimizes overfitting by creating a more abstract form of the previous feature map and thus more applicable to generic pictures of dogs. This process can be repeated for the desired number of hidden layers. Once all feature maps have been considered, the images are *flattened* and the desired output (dog or otherwise) can be generated. In many ways, CNNs are more so learning to identify small arrangements or *motifs* that resemble dogs. This concept is known as **local connectivity**, meaning two neighboring pixels are considered more relevant than two distant pixels (61). Interestingly, CNNs structurally resemble the hierarchy and pathway used by the human visual cortex found in the occipital lobe (62). Multilayer neural networks like these are also essential for the development of fully automated robots and self-driving automobiles (57).

**Figure 8:**
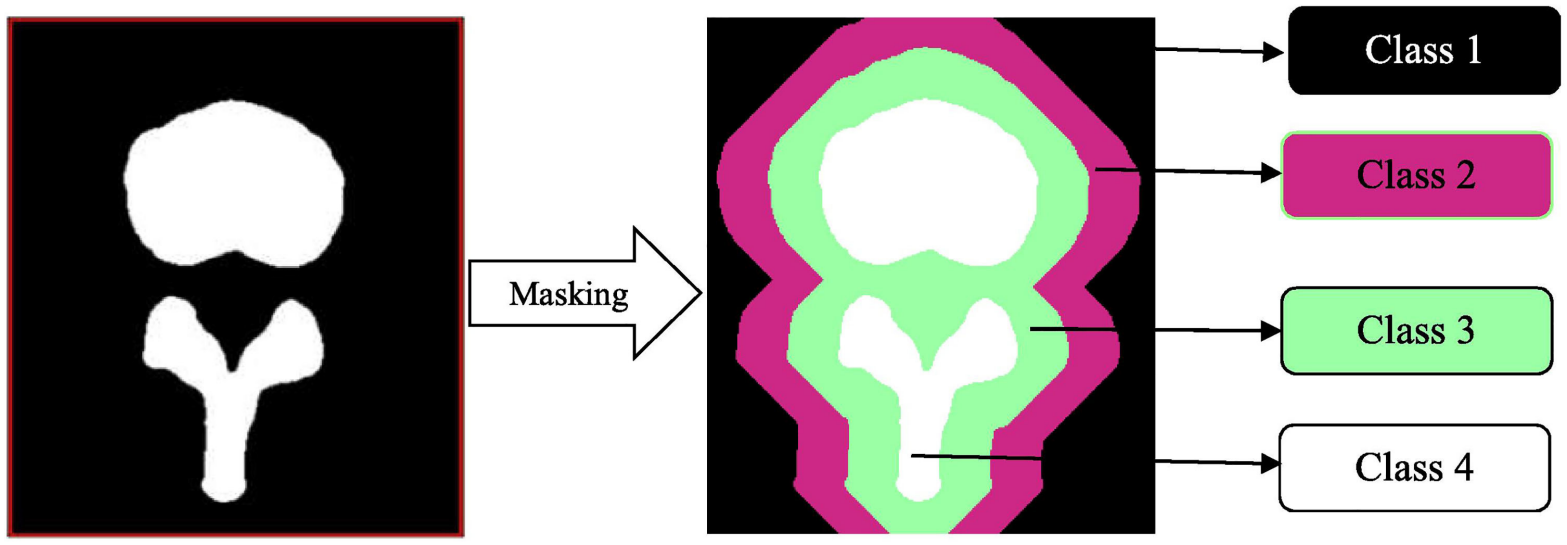
Masking in a convolutional neural network. This automated vertebral column segmentation model incorporates masking to generate two redundant classifiers for a traditionally binary classification task. Class 1 represents the background. Class 2 and 3 are unique redundant classifiers. Class 4 is the spine. By doing this, the machine becomes more adept at identifying varieties of vertebra, instead of the background. Reproduced with permission by Vania et al. (33).

In spine surgery, computer vision technology has risen in parallel with the use of computer assisted navigation, robotic surgery, and augmented reality in the operating room, all of which require high fidelity 3D reconstructions of the spinal column from computed tomography or magnetic resonance imaging scans (33, 63–67). This is achieved through automated segmentation and detection of vertebrae via ANNs. Vania et al. recently reported the results of their CNN for automated vertebral column segmentation with a unique classification system (33). Instead of the traditional classifiers of “vertebrae” vs. “not vertebrae,” they implemented four classifications (background, spine, and two redundant classifiers) as show in Figure 8 (33). They did this in order to minimize overfitting so that the learner could consider variabilities in vertebral width and length outside of the training dataset. Their model generated a sensitivity and specificity of 0.97 and 0.99, respectively, both of which were either better or comparable to other commonly applied methods (33). In addition to spinal segmentation, significant strides have also been made in automated detection of vertebral compression and posterior element fractures, as well as the grading of lumbar stenosis (18–20, 54). The potential for successful translation for preoperative and intraoperative care is promising in spine surgery. For example, automation would allow for consistent application of sagittal deformity parameters by minimizing manual measurements and displaying associated risk factors all in one software ecosystem.

**Figure 9:**
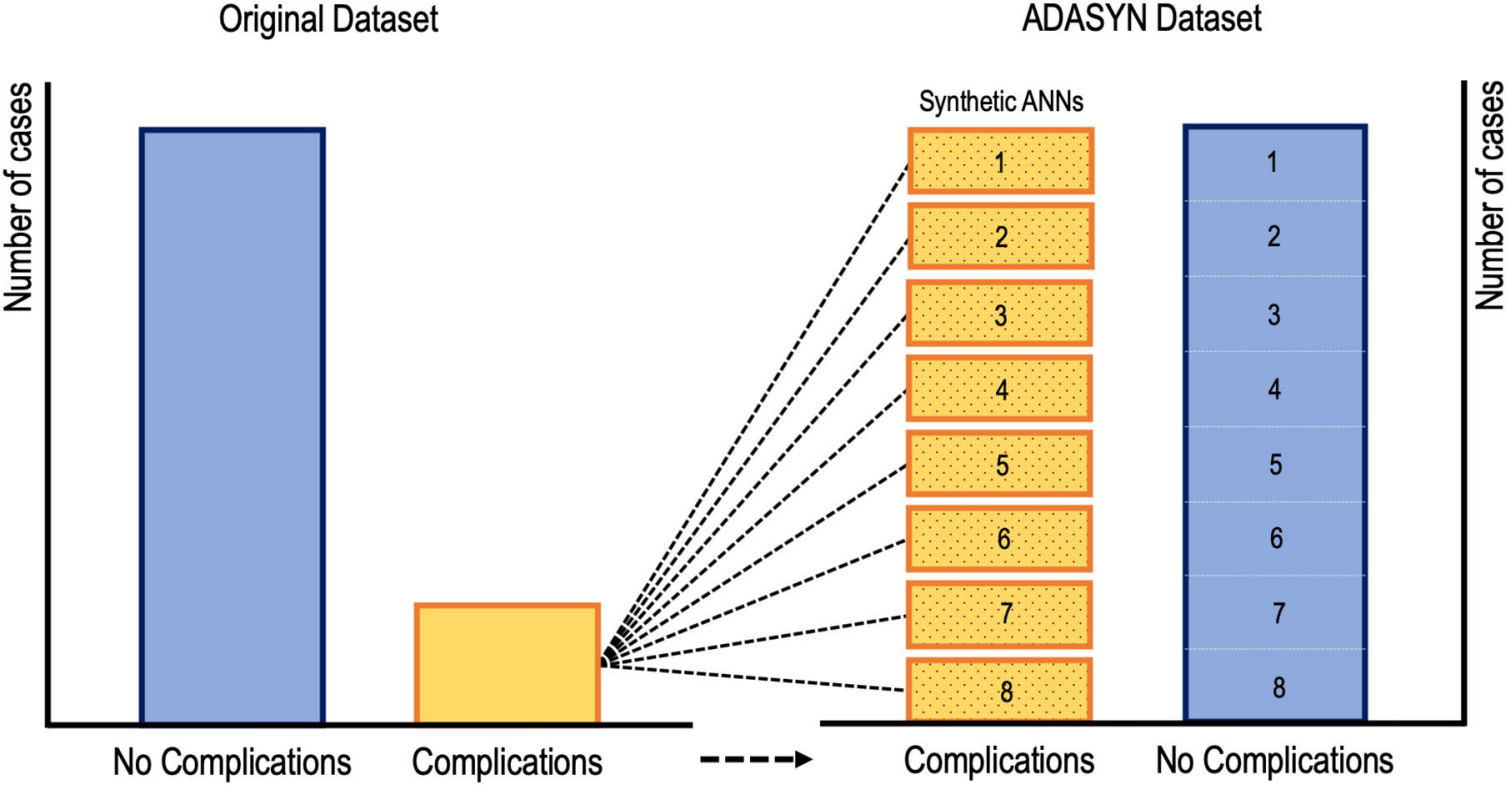
Visual representation of oversampling low incidence complications via adaptive synthetic sampling approach to imbalanced learning or ADASYN. Oversampled synthetic neural networks are created and then compared to subsets of the “no complication” cohort (68).

While supervised learners, including CARTs and SVMs, have been used to predict postoperative outcomes, there is evidence to suggest that ANNs may be the preferred method for such tasks going forward (22, 23, 27, 28, 68). Kim et al. utilized an ANN to predict cardiac and wound complications, venous thromboembolism (VTE), and mortality rates after posterior lumbar fusion from an ACS-NSQIP cohort (68). Their learner was rather informative because they addressed the problem of low complication incidence by applying ADASYN (adaptive synthetic sampling approach to imbalanced learning). As shown in Figure 9, ADASYN generates multiple synthetic cohorts with positive complications that can be compared with controls, essentially creating multiple ANNs with different weights. The final ANN achieved an AUC of 0.71 for predicting cardiac complications postoperatively, which was superior to both logistic regression and American Society of Anesthesiologists (ASA) score (68). However, the regression model proved to be superior to the ANN for predicting VTE, mortality, and wound complications. In another investigation, Hopkins et al. applied an ANN with 35 input variables on over 4,000 cases of posterior spinal fusions to predict surgical site infections (27). Their model reliably predicted both infected and non-infected cases with an AUC of 0.79 across all their neural network iterations. However, the model unexpectedly demonstrated that intensive care unit admission and increasing Charlson Comorbidity Score were *protective* against surgical site infections, both findings of which are contradictory to the literature (27). The inability to interpret what seems like inconsistent findings is a key dilemma when applying ML in clinical medicine. Though, it is possible that such associations exist in a non-linear fashion that cannot be appreciated intuitively. And while surgeon's acumen and experience must be integrated with decision support tools, there is still significant deficits in these models before they can be safely (and without hesitancy) applied when patient lives are at stake.

## Future Perspectives on Machine Learning and Spine Surgery

Machine learning and artificial intelligence are progressively becoming more commonplace in modern society. We all in some ways either actively or passively contribute to Big Data through the use of smartphones, online shopping, wearables, among other activities even unbeknown to us. Moreover, the average physician is even more “plugged-in” to the modern technological ecosystem, given the use of electronic medical records, decision support tools, and imaging software. In spine surgery specifically, the nature of dealing with vital anatomic structures in the operating room instills an eagerness for innovations that might balance operative efficiency, patient safety, and surgical outcomes. Machine learning is at the core of AI advancement in healthcare and there are definite reasons for optimism.

**Figure 10:**
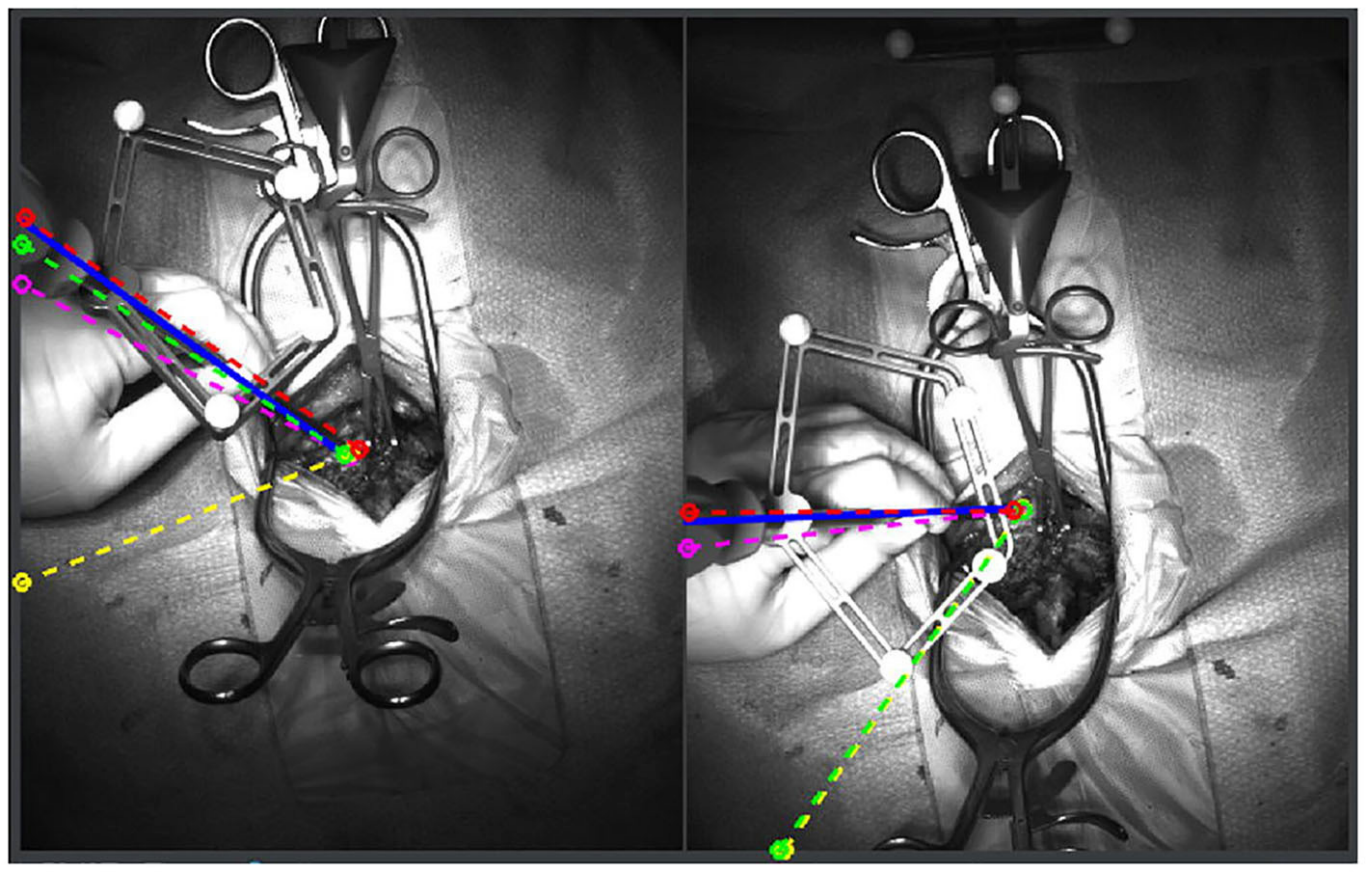
Augmented reality system that superimposes pedicle screw trajectories from computer assisted navigation onto the operating field. By minimizing the need to memorize trajectories from a separate screen, the surgeon is more readily able to identify safe zones. The blue, red, pink, yellow, and green lines represent correct, medial, lateral, superior and inferior breaches, respectively. Reproduced with permission by Nguyen et al. (69).

**Figure 11:**
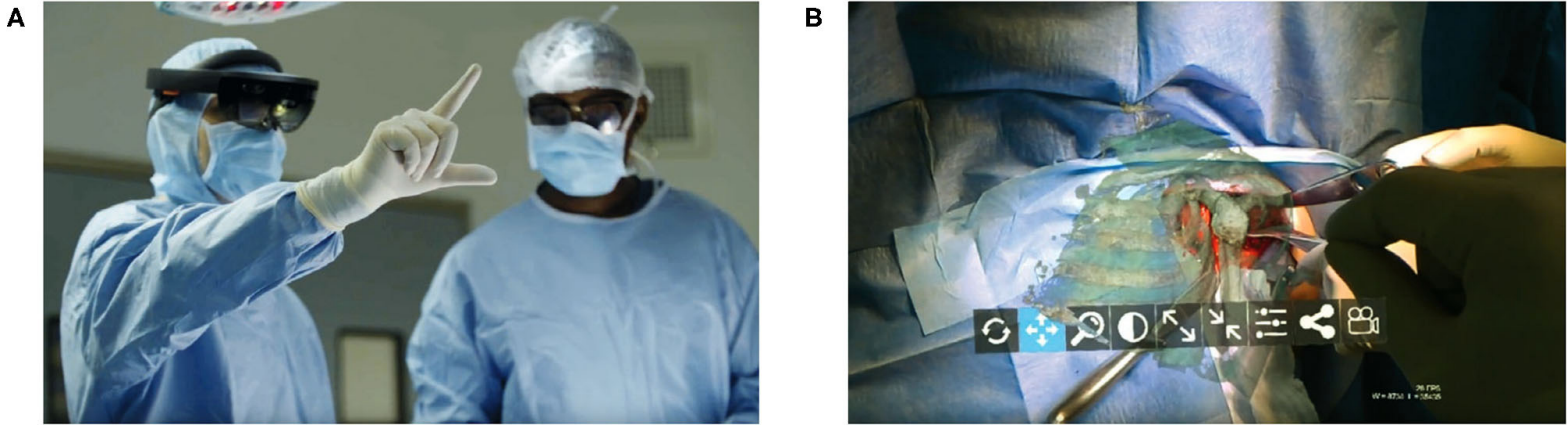
A proof of concept application of *Microsoft Hololens* for reverse total shoulder arthroplasty. **(A)** The surgeon is able to view in real-time and place in space a 3D hologram from a CT of the patient's scapula. **(B)** A 3D hologram of the patient's scapula is superimposed intraoperatively in order to fully visualize the glenoid and other relevant anatomy. Reproduced with permission by Gregory et al. (74).

As discussed previously, machine learning applications for computer vision will continue to optimize computer assisted navigation systems used by spine surgeons. AI implementation in the operating room has begun to transcend beyond what was previously possible through the use of augmented or mixed reality (69–72). Nguyen et al. in a trial of augmented reality for pedicle screw insertion with navigation, designed a virtual road map that was superimposed on the surgical site of patients undergoing spinal fusion (69). Their intention was to address the underlying obstacle of surgeons memorizing optimal screw trajectory provided by navigation, which is typically displayed away from the surgical site. By installing two overhead stereoscopic cameras, they coordinated intraoperative video with data sourced from the navigation's infrared tracking system. A representation of their innovative design is shown in Figure 10 (69). While they did not attempt to display their augmented reality system through a headset, other investigators have undertaken pilot studies as proof of concept with devices such as the *Microsoft HoloLens* (71–75). In the shoulder arthroplasty literature, Gregory et al. presented a proof of concept study using the *Hololens* to superimpose a 3D hologram of a patient's scapula in real time during a shoulder replacement (74). This application of mixed reality in the operating room was impressive because the headset did not need to be synced to a navigation system, the hologram could be adjusted in space, and the surgeon's point-of-view could be teleconferenced to others (Figure 11). Looking forward, these innovations in computer vision for the spine may also pave the way for significant improvements for surgical robots. Spine surgery robots presently appear rudimentary when compared to those utilized for minimally invasive gastrointestinal, urologic, and gynecologic surgeries. And while there is little reported on even a semi-automated robot for the spine, machine learning advancements may change this trajectory as it has for self-driving cars. However, spine surgeons (for patient safety concerns) may purposefully interact with robots in a *slave-and-master* paradigm in order to maintain total control over the machine. Using the five levels of autonomy described by the Society of Automotive Engineers, ranging from “no” to “full” automation, experts have postulated that clinical medicine may only ever incorporate up to “conditional” automation, where the machine both drives and monitors the circumstances, but humans are available for backup (9, 76).

Finally, as foreshadowed in the *Overview of Machine Learning* section, a major component of artificial intelligence research involves the ethical challenges of implementing machine learning for clinical practice (2, 4, 77–79). This has colloquially been termed the **black box**, which is the near impossible task of interpreting or explaining as to how a learner reaches the conclusions that it does, no matter how accurate it is (2, 4). And though the black box is typically attributed to ANNs and deep learning, it is also problematic for supervised learning. If a machine is learning non-linear associations in a manner that is hidden from both the engineer and the consumer, there will undoubtedly be apprehension toward the safety of an otherwise promising tool. As described by Dr. Alex John London, a professor of philosophy and artificial intelligence at Carnegie Mellon University, “the most powerful machine learning techniques seem woefully incomplete because they are atheoretical, associanist, and opaque.” As mentioned earlier in the study by Hopkins et al. for predicting surgical site infections, their neural network operated according to associations that oppose what spine surgeons consider grounded truths (27). To characterize this further, Caruana and colleagues published an infamous and equally informative machine learning model for predicting mortality after inpatient admission for pneumonia. While their learner was accurate, it reasoned that asthmatic patients with pneumonia should receive *less aggressive* care because on average they do better than non-asthmatics with pneumonia (80). This suggested course of action was in direct opposition to modern management guidelines for asthmatics, who are regularly provided the *most aggressive* care. However, Caruana et al. learner was not attuned to such contextual guidelines. Thus, from a prediction standpoint, asthmatics with pneumonia in an intensive care unit were observed (by the model) as experiencing better outcomes relative to the general population that is treated more conservatively. This harkens back to the point previously discussed regarding the importance of understanding exactly which question the model is being asked to answer. Beyond the black box, other ethical and logistical obstacles in machine learning in medicine include **distributional shift** (training datasets that may be biased toward race or socioeconomic status or simply outdated), **insensitivity to impact** (predictive tools that underestimate the consequences of a false positive or false negative outcome), and **reward hacking** (the machine learns unexpected means of achieving an outcome that cheat the system) (4).

While the challenge of explaining machine learning's method for reasoning persists, it draws some similarities to the way clinical medicine is practiced in the present. Physicians, much like deep learners, often treat patients using some component of their clinical experience or *gestalt* (difficult to explain) in addition to their technical knowledge (easy to explain). And the solution to this problem may involve a combination of (1) accepting the black box of machine learning, and (2) testing them rigorously against multiple patient cohorts (79). Altogether, these examples from the literature suggest the need for a healthy level of skepticism toward machine learning, and a willingness to appreciate its methodology.

## Author Contributions

MC and NP were responsible for reviewing publications for inclusion in the review, drafting of the manuscript, and creating table and figures for the manuscript. JC and KN were responsible for critical revision of the manuscript for important intellectual content related to machine learning methodology and spine surgery research. AV was responsible for the conception of the review, supervision, and critical revision of the manuscript for important intellectual content. All authors contributed to the article and approved the submitted version.

## Conflict of Interest

The authors declare that the research was conducted in the absence of any commercial or financial relationships that could be construed as a potential conflict of interest.
